# Contemporary evidence to inform management of deceased potential thoracic organ donors after brain death

**DOI:** 10.1016/j.jhlto.2025.100474

**Published:** 2025-12-29

**Authors:** Federico Ciardi, Chiara Magri, Antonio Rubino, Emily A. Vail

**Affiliations:** a Department of Anesthesiology, Columbia University Irving Medical Center/New York-Presbyterian Hospital, New York, NY; b Department of Anesthesia, Critical Care and Emergency, Fondazione IRCCS Ca' Granda Ospedale Maggiore Policlinico, Milan, Italy; c Department of Cardiothoracic Anaesthesia and Intensive Care, Royal Papworth, Cambridge, UK; d Department of Anesthesiology and Critical Care, University of Pennsylvania Perelman School of Medicine, Philadelphia, PA

**Keywords:** Brain death, Thoracic transplantation, Donor management, Critical care, Organ procurement, Evidence-based practice

## Abstract

Brain death triggers profound physiologic derangements that threaten organ viability and contribute to the persistent shortage of transplantable thoracic organs. This narrative review synthesizes contemporary evidence informing the management of deceased potential thoracic organ donors after brain death, focusing on clinical studies published over the past decade. We identified 14 trials addressing key aspects of donor management, including lung-protective ventilation strategies, prone positioning, targeted temperature management, hormonal supplementation, and cardiovascular support. Notable findings include validation of lung-protective ventilation with higher positive end-expiratory pressure improving lung recovery rates, observational evidence suggesting that prone position is associated with increased lung and heart procurement, and recent randomized trials demonstrating no benefit from routine levothyroxine administration even in hemodynamically unstable donors. Corticosteroid therapy appears to effectively reduce vasopressor requirements, while newer evidence supports the safety of lower-dose regimens with improved glycemic control. Despite these advances, significant knowledge gaps persist regarding optimal hemodynamic monitoring, vasopressor selection, and other fundamental management decisions. The fragmented nature of the donation and transplantation system presents unique challenges for evidence generation and implementation. Ongoing developments, including donor care units, international collaboration, and innovative trial designs, offer opportunities to address these gaps. This review provides intensivists with an evidence-based framework for optimizing donor management while highlighting priority areas for future research to increase the availability and quality of thoracic organs for transplantation.

## Background

Organ transplant is the last line of therapy for select end-stage diseases. As the population grows and ages, the demand for organs continues to increase. However, the relative stagnation in the worldwide availability of thoracic organs results in an unmet and growing health need.^1^, ^2^ Over the past 10 years, thoracic organ recovery rates from consented deceased organ donors have remained stubbornly lower than those for extrathoracic organs. While scientific innovation in organ recovery and preservation promises to expand access to thoracic organs donated after circulatory death,^3^ recent clinical trials in donors after brain death (DBD) suggest that some pre-recovery interventions may further increase availability and quality of organs for transplant. While the conduct of interventional research in deceased potential organ DBD requires careful navigation of inherent practical and ethical challenges,^4^, ^5^ the recent publication of high-quality clinical trials in major biomedical journals^6^, ^7^, ^8^, ^9^ illustrates their feasibility.

Since the 2020 publication of DBD management guidelines by the ISHLT^10^ and the Canadian Critical Care Society,^11^ clinical evidence has continued to accumulate. Therefore, this manuscript aims to summarize recent clinical research informing the management of deceased potential organ DBD and to identify knowledge gaps and opportunities to improve donor management through evidence-based practice. Acknowledging the breadth of therapies and outcomes studied, this narrative review will focus on clinical studies published in English over the past decade. Studies included potential thoracic organ donors who received interventions between the declaration of brain death and organ recovery surgery and reported physiologic, donation, or recipient outcomes. Using a framework familiar to intensivists, evidence is organized by organ systems.

## Foundations of clinical donor management

Potential DBD donors require intensive management of complex clinical problems, including the comorbid disease, the pathophysiologic consequences of brain death, and concomitant injury or illness.^12^, ^13^ Donor management is particularly challenging when clinicians’ or hospitals’ clinical experiences are limited: the incidence of donation after brain death is very low outside of dedicated donor care units, high-volume trauma centers, or neurosurgical specialty hospitals.^14^ Given known limitations in clinician experience, clinical practice guidelines may address knowledge gaps, standardize care, and improve donation outcomes.

Globally, health systems in different countries organize procurement, donor management, and organ allocation in various manners. Many European systems, such as France and the United Kingdom, operate centralized governmental donation networks that prioritize donor autonomy, nonmaleficence and delegate clinical decision-making to the treatment team at the intensive care unit of the donor’s death. Other countries involve non-governmental agencies in all aspects of the process, which concentrate expertise on this complicated and often clinically challenging scenario to optimize organ quality and yield. These range from strictly nationally regulated and governed systems (United States, Canada, South Korea, and Spain) to the low- and middle-income country model led by private sector involvement (e.g., South Africa, India, Ethiopia, and Mongolia). Variable system designs often reflect the ethical values, laws, and healthcare system infrastructure of the countries in which they operate.

While available clinical donor management guidelines have well-described limitations,^15^ they provide practical frameworks for assessing the quality of available evidence and identifying areas of need and potential focus. Over the past 10 years, three guidelines have been published by US and Canadian professional societies.^10^, ^11^, ^16^ Table 1 outlines organ-specific recommendations made by each guideline to illustrate areas of agreement and discrepancies between recommendations.

**Table 1:** Comparison of Deceased Potential Donor After Brain Death Management Recommendations Between 3 Guidelines/Consensus Statements Published 2015-2020

		SCCM/ACCP/AOPO consensus statement 2015^16^	JHLT consensus statement 2020^10^	Canadian council for donation and transplantation 2020^11^
	Hemodynamic monitoring	PAC, CVC, NICOM should be considered	Use routine arterial line and CVC PAC if hypotensive	No routine PAC
	Echo-cardiography	Use TTE as preferred test Use TEE if TTE is inconclusive or cannot be performed adequately Timing: defer until donor has weaned off catecholamines. Repeat every 12-24 hours	Use TTE or TEE for all potential heart donors Timing: repeat every 12 hours	Use TTE or TEE for all potential heart donors Timing: consistent with general ICU practise
	Angiography	If donor >40y or has risk factors for CAD	If donor has risk factors for CAD	If donor has risk factors for CAD
	Hemodynamic target	MAP > 60 mmHg UOP > 1 ml/kg/hr LVEF >45% Lower vasopressor dose	MAP >60 mmHg CVP 6-10 mmHg UOP 0.5-2.5 ml/kg/hr	MAP>65 mmHg
	Arrhythmia management	Consider short acting agents	If tachyarrhythmias with hemodynamic instability, consider synchronized cardioversion. If bradycardia consider chronotropic agent (epinephrine, dopamine, isoproterenol or dobutamine)	
	Resuscitation fluids	Use crystalloids (0.9% saline and Lactated Ringer’s solution) or colloids Avoid hydroxyethyl starch	Use crystalloids (0.9% saline or Lactated Ringer’s solution) If hypernatremia consider dextrose-containing fluids or hypotonic solutions. If metabolic acidosis consider sodium bicarbonate	Use crystalloids rather than colloids
	Ventilation strategy	Consider ventilator strategies utilizing low stretch protocols and measures to recruit atelectatic lungs	V_t_ 6-8 ml/kg IBW PEEP 8-10 cmH_2_O PIP < 35 cmH_2_O pH 7.35-7.45 PaCO_2_ 35-45 PaO_2_ 80-100, SaO_2_ > 95%	V_t_ 6-8 mg/kg IBW PEEP 8 cmH_2_O
	Recruitment maneuvers		After disconnection of the ventilator circuit	After disconnection of the ventilator circuit
	Broncho-scopy	Use for diagnosis and treatment in all potential lung donors	Use for diagnosis and treatment in all potential lung donors Repeat at procurement, compare findings to previous studies	Use for diagnosis and treatment in all potential lung donors
	Other		Consider SABA/LABA bronchodilators if needed. Daily CXR Additional chest imaging if indicated 30° head of bed elevation	No routine bronchodilators Single routine CXR Additional chest imaging if indicated
	AVP deficiency	If hypotensive with low SVR, start *iv* AVP at 0.01-0.04 IU/min If normotensive but DI with hypernatremia (sodium > 145-150 mmol/L) start desmopressin. If hemodynamically unstable, use both AVP and desmopressin	If hypotensive, start *iv* AVP titrated to UOP <3 ml/kg/h and improve MAP. If normotensive, use desmopressin	If hypotensive, use AVP If normotensive, use desmopressin
	Serum sodium target	130 and 150 mEq/L	135-150 mEq/L	135-155 mEq/L
	Other electrolytes	Other electrolytes should be monitored and replenished if necessary		
	Steroids	Methylprednisolone 1 g *iv*, 15 mg/kg *iv* or 250 mg *iv* bolus followed by infusion at 100 mg/hr should be administered after blood has been collected for tissue typing	Use methylprednisolone 15 mg/kg *iv* bolus after brain death	Use intravenous corticosteroid therapy for donor requiring vasopressor support
	Thyroid hormone	Should be considered for hemodynamically unstable donors or LVEF <45% Both T_3_ and T_4_ are acceptable for use	May be considered for managing LVEF <45% or hemodynamically unstable donors, but efficacy remains uncertain	No routine thyroid hormone supplementation. No recommendation for patients with hemodynamic instability or cardiac dysfunction
	Blood products	Hb goal >7g/dL No FFP or platelets without bleeding		Hb goal >7.0 g/dL No FFP without bleeding No platelets until <10 × 10^9^/L
	GI	Give enteral nutrition Intestinal decontamination and decompression for small bowel donors	Give enteral nutrition or TPN	Give enteral nutrition
	Glycemia	Routine management of hyperglycemia (<180 mg/dL) Avoid dextrose-containing crystalloid solutions	Blood glucose target 72-180 mg/dL	Blood glucose target 108-180 mg/dL Check Hb A1c for pancreas donors
	Antibiotic therapy	Antibiotic treatment if necessary	Antibiotic treatment if necessary Prophylactic use is not clinically warranted	Antibiotic treatment if necessary
	Temperature		36-37 °C	34-35 °C unless kidneys will not be recovered

ACCP, American College of Chest Physicians; AOPO, Association of Organ Procurement Organizations; AVP, arginine vasopressin; CAD, coronary artery disease; CO, cardiac output; CVC, central venous catheter; CXR, chest X-ray; DI, diabetes insipidus (also arginine vasopressin deficiency); FFP, fresh frozen plasma; Hb, hemoglobin; ICU, intensive care unit; JHLT, Journal of Heart and Lung Transplantation; LABA, long-acting beta-agonist; LVEF, left ventricular ejection fraction; MAP, mean arterial pressure; NICOM, noninvasive cardiac monitor; PAC, pulmonary artery catheter; PaCO2, arterial partial pressure of carbon dioxide; PaO2, arterial partial pressure of oxygen; PAOP, pulmonary artery occlusion pressure; PEEP, positive end-expiratory pressure; PIP, peak inspiratory pressure; RBC, red blood cells; SABA, short-acting beta-agonist; SCCM, Society of Critical Care Medicine; SVR, systemic vascular resistance; T3, triiodothyronine; T4, thyroxine; TEE, transesophageal echocardiography; TPN, total parenteral nutrition; TTE, transthoracic echocardiography; UOP, urine output; Vt, tidal volume.

## New and emerging evidence by organ system

### Pulmonary

#### Ventilation strategies

Fifteen years ago, a small, randomized trial published by Mascia et al^17^ demonstrated that a lung protective ventilation strategy (including higher positive end-expiratory pressure (PEEP), lower tidal volumes and driving pressure, and measures to prevent and treat atelectasis) could improve both the eligibility and availability of lungs for transplantation from potential DBD donors. Since then, three other studies have been published, all aiming to refine donor-specific ventilation strategies.^9^, ^18^, ^19^

First, Miñambres et al (2016) published a before-and-after observational study that evaluated the change in lung and other organ recovery rates and graft function before and after the implementation of an intensive lung donor protocol.^18^ The protocol included lung-protective ventilation, hourly recruitment maneuvers, frequent bronchoscopy, hemodynamic management, and high-dose methylprednisolone. Although the lung donation rate among the intervention cohort was significantly lower than in other contemporaneous studies, investigators demonstrated that the intensive lung donor treatment protocol was associated with a doubling of the lung procurement rate without impacting the yield or quality of other organs or lung recipient outcomes.

Second, Marklin et al (2021) evaluated prone ventilation in DBD donors with hypoxemia (P/F ratio < 300) and atelectasis.^19^ This non-randomized, observational cohort study compared 40 donors receiving prone positioning for ≥12 hours with 79 historical controls. Prone ventilation was associated with improved P/F ratios and increased lung transplantation by 21% (*p* = 0.03) and heart transplantation by 23% (*p* = 0.015). Recipients of lungs from prone donors showed better P/F ratios at 72 hours (mean difference +129, *p* = 0.08), though 3-month survival was similar. This intervention is resource-intensive and can be associated with complications, especially in intensive care units unfamiliar with advanced management of respiratory failure. Adverse effects were not recorded in this study. Despite baseline differences between groups and temporal confounders, these findings align with intensive care unit evidence supporting prone positioning for lung recruitment.

Most recently, Ware et al (2025) aimed to evaluate whether an “open lung protective ventilation” strategy (tidal volume 8 ml/kg, PEEP 10 cmH_2_O, protocolized recruitment maneuvers every 8 hours) improved lung utilization for transplantation compared to conventional ventilation with larger tidal volumes and lower PEEP.^9^ Neither the primary outcome of lung utilization for transplantation nor secondary outcomes (including changes in PaO_2_/FiO_2_ ratio, need for vasopressors, and serious adverse events) were different between the two groups. Despite a well-designed, prospectively randomized interventional trial, the study was terminated early due to external circumstances, and outcomes were available for only 18% of recipients, highlighting the challenges of linking donor management interventions to recipient outcomes.

### Cardiovascular

As brain-dead potential organ donors commonly develop multifactorial hypotension, myocardial dysfunction, and arrhythmias, available donor cardiovascular system guidelines include recommendations for hemodynamic monitoring, hemodynamic goals, arrhythmia treatment, and heart graft function assessment.

#### Vasopressors

The overwhelming sympathetic outflow associated with brainstem death results in a significant increase in circulating catecholamines. This 'sympathetic storm' induces marked vasoconstriction, leading to hypertension, tachycardia, and a secondary rise in myocardial oxygen demand. Combined with catecholamine-induced coronary vasoconstriction, this results in a myocardial oxygen supply-demand imbalance and consequent subendocardial ischemia. These mechanisms contribute to the early cardiac dysfunction in brain-dead donors. The initial phase of heightened autonomic activity is followed by catecholamine depletion, which leads to profound vasodilation and a decrease in systemic vascular resistance. Therefore, hemodynamic instability in brain death can be attributed to the concurrent presence of two distinct dysfunctions: myocardial and vascular.^20^

Despite guideline-endorsed choices of vasopressor and inotropic medications, optimal strategies remain uncertain. While numerous observational studies have been published on this topic, we did not identify any new interventional trials to inform uniform selection of one medication over others. In the absence of high-quality evidence specific to potential DBD donors, intensivists may choose to apply general hemodynamic management strategies used in critically ill patients,^15^, ^21^, ^22^, ^23^, ^24^, ^25^ with some exceptions.^26^

Although dopamine has been considered a first‑line inotropic agent in donor management (e.g., according to the Society of Critical Care Medicine/American College of Chest Physicians/Association of Organ Procurement Organizations Consensus Statement 201516), accumulating evidence demonstrates that it is not superior to norepinephrine in the management of shock and may even be associated with a higher risk of arrhythmic events.^22^ Furthermore, in a subgroup of patients with cardiogenic shock, dopamine use has been linked to increased mortality.^22^ Moreover, a recent panel of ESICM experts recommends dobutamine as the preferred first‑line inotrope in patients with cardiogenic shock, while discouraging the use of dopamine.^27^

Vasopressin becomes deficient in 90% of brain-dead patients, and its absence is associated with hypovolemia and hypotension.^13^ For this reason, it is considered the first-line agent in potential donors. Conversely, the Surviving Sepsis Campaign guidelines recommend its use only as a second-line agent. Indeed, 2 independent studies^28^, ^29^ do not demonstrate a clear superiority of vasopressin over norepinephrine but reported its “catecholamine-sparing” effect: as such, the early use of vasopressin in combination with norepinephrine may help reduce the adrenergic burden associated with traditional vasoactive agents.

Table 2 provides a summary of the indications for the use of different vasopressors in the management of potential organ donors and compares these with their recommended use in septic and cardiogenic shock.

**Table 2:** Comparison of Vasoactive Medication Recommendations in Guidelines and Consensus Statements Between Resuscitation and Brain-Dead Donor Management

Current evidence in resuscitation			Current evidence in organ donors management		
Surviving sepsis campaign 2021	AHA contemporary vasopressor and inotrope use in cardiogenic shock 2023		SCCM/ACCP/AOPO consensus statement 2015	JHLT consensus statement 2020	Canadian council for donation and transplantation 2020
Second line agent if MAP inadequate	Indicated for refractory vasoplegic shock	VASOPRESSIN	Recommended as first line in cases of refractory shock	Recommended as first line agent	First line vasoactive agent for hypotension (very low evidence)
First-line agent	Indicated for CS with hypotension and vasoplegic shock	NOREPINEPHRINE	Recommended in the predominantly vasodilatory component of shock	May be used to achieve desired hemodynamic goals but may affect heart’s contractility after transplantation	Second line agent for hypotension not responding to vasopressin alone (very low evidence)
	Indicated for hypotension (vagally mediated), hypotension with severe aortic stenosis or obstructive hypertrophic cardiomyopathy	PHENYLEPHRINE	Recommended in the predominantly vasodilatory component of shock		
Consider when norepinephrine is not available (caution in patients at risk for arrhythmias)		DOPAMINE	First-line agent for cardiac pump dysfunction (insufficient data to recommend this over other vasopressors)	May be considered as first line inotropic agent	No indication in any case
Consider if cardiac dysfunction with persistent hypoperfusion	Indicated for cardiogenic shock with hypotension, vasoplegic shock, and bradycardia	EPINEPHRINE	May be used in primary cardiac pump dysfunction	May be used to achieve desired hemodynamic goals but may affect heart’s contractility after transplantation	
Consider if cardiac dysfunction with persistent hypoperfusion	Indicated for CS with preserved blood pressure (decompensated heart failure, low cardiac output state)	DOBUTAMINE	May be used in primary cardiac pump dysfunction		

ACCP, American College of Chest Physicians; AHA, American Heart Association; AOPO, Association of Organ Procurement Organizations; CS, cardiogenic shock; JHLT, Journal of Heart and Lung Transplantation; SCCM, Society of Critical Care Medicine.

#### Protocolized fluid management

As optimizing donors’ volume status may prevent end-organ injury caused by malperfusion or venous congestion, the MOnITOR trial reported protocol-guided fluid management using lithium thermodilution cardiac output monitors.^30^ 550 potential donors from eight different US donation regions were randomized to algorithm-guided fluid resuscitation or usual care (which was not standardized across donation regions). The trial was significantly limited by high dropout in the intervention arm. However, even the per-protocol analysis did not demonstrate a statistically significant difference in the primary outcome of the mean number of organs transplanted per donor.

Device-guided fluid management was further studied by Marklin and colleagues in two related manuscripts. In the original observational cohort study, investigators compared 64 consecutive donors who received a stroke volume-guided fluid resuscitation and vasopressor management protocol to 30 historical controls who received usual care.^31^ While the intervention was associated with greater fluid administration and shorter duration of vasopressor use, organ yield and quality outcomes were inconsistently different between groups. In a second manuscript, authors compared stroke volume-guided resuscitation using arterial pressure waveform pulse contour analysis against a non-invasive bioreactance monitor.^32^ A lack of spontaneous breathing in patients with brain death may improve the accuracy of stroke volume measures by bioreactance over other critically ill groups. Despite this theoretical advantage, there were no significant differences in treatment or outcome between groups among 158 prospectively enrolled DBD donors.

Collectively, these studies demonstrate that protocolized hemodynamic monitoring may be difficult to implement across centers, and its use may not improve organ yield or quality. Despite this, a recent trial of levothyroxine administration suggests that noninvasive hemodynamic monitors and pulse contour analysis systems are already in widespread use among participating US donation regions.^6^

#### Statins

Statins may protect against ischemia-reperfusion injury by reducing vascular inflammation.^33^ Nykänen et al conducted a randomized controlled trial of a single 80 mg dose of simvastatin versus no statin at the time of donor heart acceptance; the primary efficacy endpoint was postoperative plasma troponin T and I levels during the first 24 hours after heart transplantation, while secondary endpoints included post-transplant hemodynamics, inflammation, allograft function, rejection treatments, and mortality.^34^, ^35^ Recipients of simvastatin-treated hearts demonstrated a significant reduction in serum troponin, a decrease in serum NT-proBNP from 1 to 4 weeks post-transplant, and a 53% reduction in the number of treatments for acute rejection within the first 30 days. There were no differences in most recipient clinical outcomes, possibly because the study was not adequately powered to detect these differences. Recently published results of the 5-year follow-up suggest that there were no additional differences between groups beyond the initial 30 days post-transplant.^34^ Ultimately, administering a single dose of simvastatin to donors appears to be a low-risk intervention that may significantly improve allograft quality through anti-inflammatory pathways.

### Renal

#### Renal replacement therapy

Renal replacement therapy, although commonly used in other critically ill patients, was historically rarely initiated in potential organ donors.^36^ A 2022 observational study by Marklin et al compared brain-dead donors with oliguric acute kidney injury who were treated with continuous renal replacement therapy with historical donor controls.^37^ Potential donors who received continuous renal replacement therapy demonstrated longer time from declaration of brain death to organ recovery, better lung function, improvements from baseline LV ejection fraction (allowing transplantation of previously ineligible hearts), and donated more organs for transplant than those with AKI and no renal replacement. A subsequent retrospective observational study, using US deceased donor and kidney recipient registry files, was conducted by Wen et al (2024)^36^: investigators identified higher rates of delayed kidney graft function among recipients of donor kidneys with AKI and dialysis. Still, they found no differences in longer-term kidney graft or recipient survival outcomes compared to matched controls. These studies suggest that initiating renal replacement therapy after brain death is feasible. Despite inherent selection bias in these study designs, renal replacement for deceased potential donors may improve both lung and heart yield through fluid balance optimization and extended evaluation time. Randomized trials are still required to validate these findings.

#### Targeted temperature management

Studies of therapeutic hypothermia demonstrate a fundamental challenge of clinical donor management research: interventions targeting 1 organ system can have unintended consequences on others. In a randomized interventional trial of 370 deceased potential DBD, Niemann et al (2015) demonstrated that controlled hypothermia (34 °C-35 °C) from the time of brain death declaration reduced delayed graft function in kidneys, with a greater benefit in extended-criteria donors.^8^ Importantly, they found no difference in recovery rates for other organs. However, two follow-up studies suggested that donor hypothermia was associated with impaired donor heart function.^38^, ^39^ These findings highlight the limitations of single-organ focused interventions in the context of multi-organ donation, where trials are often powered for one organ endpoint and may overlook unintended effects on other organs. Given this uncertainty, as well as newer studies demonstrating superior kidney graft outcomes using post-recovery hypothermic machine perfusion,^40^ current evidence supports maintaining normothermia in DBD donors. Given the loss of hypothalamic function associated with brain death, normothermia may require active warming interventions. The evolution of evidence in deceased donor temperature management also illustrates how the introduction of new technologies (specifically, advances in organ preservation) may ultimately simplify donor management and improve multi-organ outcomes.

### Endocrine

#### Steroids

Following brain death, hypothalamic-pituitary-adrenal axis disruption often leads to adrenal insufficiency and hypotension.^41^ While corticosteroid administration may reduce vasopressor requirements, its impact on transplant outcomes remains unclear.^42^, ^43^ In 2022, Van Bakel et al published a factorial RCT comparing high-dose methylprednisolone, levothyroxine, combination therapy, and no hormonal treatment in 199 brain dead donors.^7^ Methylprednisolone (alone or in combination) significantly reduced the donor vasoactive-inotropic score (the primary outcome); however, levothyroxine alone showed no benefit. Organ yield was similar across groups, although levothyroxine alone was associated with a 20% lower rate of lung recovery. These findings align with earlier RCT data by Venkateswaran et al (2008), which demonstrate reduced extravascular lung water after steroid therapy.^44^

Corticosteroid doses varied widely across studies included in recent meta-analyses^42^, ^43^: The first observational cohort trial directly comparing dosing regimens showed that lower-dose hydrocortisone (300 mg *iv* once followed by 100 mg *iv* every eight hours) was non-inferior to high-dose methylprednisolone (15 mg/kg *iv* once or repeated after 24 hours) in both donor hemodynamics and organ yield.^45^ In that study, insulin requirements were lower in the lower-dose hydrocortisone group. Given current evidence, a low-dose hydrocortisone regimen appears reasonable to support donor stability while avoiding known side effects.

#### Thyroid hormone replacement

Focusing specifically on the common practice of thyroid hormone supplementation to treat myocardial dysfunction and hypotension attributed to acute thyroid insufficiency after brain death, Dhar et al (2023) published one of the highest-quality trials to date.^6^ A multicenter, open label randomized controlled non-inferiority trial involving 838 brain-dead donors requiring vasopressors who received either intravenous levothyroxine infusion or placebo. The primary outcome, transplantation of donor hearts, was identical between groups. There were no significant differences in secondary outcomes (graft survival at 30 days, vasopressor weaning, donor ejection fraction, and organs transplanted per donor). However, adverse events such as severe hypertension and tachycardia were more frequent in the levothyroxine group. These findings reinforce Van Bakel et al’s results,^7^ suggesting that thyroid hormone replacement offers no benefit, even in hemodynamically unstable donors or those with myocardial dysfunction, and may cause harm. In conclusion, high-dose methylprednisolone significantly reduces vasopressor requirements in brain-dead organ donors, whereas levothyroxine alone does not provide a similar benefit.

#### Glycemic control

Contemporary clinical guidelines for potential DBD recommend management strategies like those used in other critically ill adults. Aljiffry et al (2016) reported a small randomized controlled pilot study of 15 potential donors applying a novel hyperinsulinemic normoglycemic physiologic “clamp” technique designed to improve the metabolic conditioning of liver grafts and reduce systemic inflammation.^46^ The study found improvements in glycemic control and systemic inflammation, though it was underpowered to detect any clinically significant differences between groups.

### Hematology

While recent donor management guidelines recommend transfusion thresholds for packed red blood cells and platelets like those of other critically ill populations, there is a paucity of interventional trials in hematologic management of DBD donors, with no new prospective evidence published in the last decade. Two retrospective observational studies have analyzed blood product transfusion thresholds in DBD donors.^47^, ^48^ Taken together, these studies support a restrictive transfusion threshold (<7 g/dL); there is no evidence that higher thresholds improve organ function or procurement rates.

While commonly administered in other critically ill patients, we did not identify any studies examining the delivery or efficacy of routine venous thromboembolism prophylaxis in potential organ donors.

### Protocolized donor management

Given the known complexity of donor management, many hospitals and organ procurement organizations have developed comprehensive local protocols for bedside clinicians. Malinoski et al have published multiple observational studies demonstrating associations between meeting a set of “donor management goals” (clinical and laboratory endpoints used as both descriptors of donor severity of illness and measures of donor management quality) and higher organ yield.^48^, ^49^, ^50^ In the United Kingdom, a structured “donor optimization bundle” has been developed by NHS Blood and Transplant to guide the management of potential DBD donors.^51^ Building on this idea, Westphal et al (2023) evaluated the efficacy of an evidence-based checklist for brain-dead potential donor management in 1535 patients across 63 Brazilian hospitals.^52^ They found no significant reduction in the primary outcome of cardiac arrest before the organ recovery procedures. However, the subgroup with the highest checklist adherence (>79%) had significantly lower rates of cardiac arrest (5.3% vs 14.8%, *p* = 0.006) and higher organ yield. This represents the first large-scale cluster randomized trial testing goal-directed donor management protocols, with findings suggesting that checklists may be challenging to implement across institutions but may improve donor management quality (as defined by prevention of serious adverse events).

## Discussion

This narrative review of clinical trials published over the past decade illustrates enthusiasm for innovative management of deceased thoracic organ DBD, with the potential to improve outcomes for both deceased organ donors and recipients. Building on foundational evidence compiled in recent clinical donor management guidelines, this narrative review identified 12 new clinical studies of relevance to intensivists managing deceased potential organ DBD. This work may strengthen recommendations already captured in some guidelines, such as lung-protective ventilation. New studies also call into question the ongoing use of 2 previously recommended interventions-routine administration of levothyroxine and hypothermia before organ recovery.

### Identified gaps

Amidst these modest gains, evidence remains incomplete. We found little to no new interventional evidence to inform fundamental donor management practices, including hemodynamic monitoring modalities (pulmonary artery catheters versus noninvasive cardiac output monitors) and choice of vasopressors. Furthermore, no high-quality studies have been performed to establish specific hemodynamic or electrolyte targets, preferred resuscitation fluids, or blood product transfusion thresholds that differ from standard critical care practice. Other routine interventions lacking donor-specific evidence include venous thromboembolism prophylaxis, nutritional support (enteral versus parenteral), and glycemic control targets (outside of studies of multiple co-administered interventions).^48^, ^49^, ^50^ This absence of evidence for fundamental management decisions highlights the continued reliance on extrapolation from general critical care principles rather than the availability of donor-specific information.^53^

Recommendations regarding prone positioning and statin administration cannot yet be made given the low level of evidence available to date. To this end, we look forward to the results of two randomized trials in progress to further inform these practices; 1) a pilot randomized controlled trial at the University Health Network, Toronto, comparing donor prone ventilation versus standard of care (NCT06259357) and 2) the UK National Health Service Blood and Transplant's multicenter simvastatin trial enrolling 2600 donors across 89 sites.^54^ In the meantime, given the clinical problems and therapies shared by potential deceased organ donors and other critically ill patients, intensivists may consider using established and evolving critical care management practices in both groups.^53^ While awaiting definitive evidence generation in donors, adopting proven interventions from living critically ill patients and systematically monitoring for unintended consequences ensures that donors are managed according to contemporary best practices, with the hope that doing so may minimize common complications and preserve opportunities for organ and tissue donation.

### Challenges and barriers

The unique challenges inherent to conducting clinical research in deceased potential organ donors and transplant recipients represent one significant barrier to evidence generation. These challenges include obtaining informed consent for research from donor families at a time of unexpected stress and grief, as well as ethical concerns about the ability of transplant programs and waitlist patients to consent to participation without coercion.^4^, ^5^ Second, deceased potential organ donors are heterogeneous, spanning different ages, death etiologies, and comorbidity profiles within and between countries, introducing challenges for study power and generalizability between donation systems. Third, some interventions designed to optimize one organ may harm others (as illustrated by studies of donor hypothermia above).^8^, ^23^, ^38^, ^39^, ^40^ Fourth, interventions may impact a range of outcomes (from physiological donor endpoints, organ acceptance, donation, to short- and long-term graft and recipient outcomes)—all of varying importance to patients and other transplant system stakeholders. A lack of defined core outcomes for organ donor research also hinders the coherent synthesis of evidence from studies of similar interventions. Fifth, system fragmentation severely impedes research conduct and assessment of outcomes. Separation of donor hospitals’, organ procurement organizations’, and transplant centers’ data systems) limits investigators’ abilities to reliably link donor management processes and recipient outcomes in retrospective and prospective research.^55^ Investigators’ lack of access to recipient data is illustrated starkly in the Ware study, where outcomes were unavailable for 80% of lung transplant recipients.^9^ Finally, the rapidly evolving landscape of post-mortem perfusion technologies raises questions about whether optimizing in situ donor management remains relevant when organs can be further evaluated and rehabilitated during and after recovery.^40^

Despite the appearance of new evidence, the unmet need for transplantable organs remains a pressing concern, particularly for thoracic organs.^1^, ^56^ One contributing factor may be a persistent but largely uncharacterized translation gap between scientific discoveries and clinical application. While several studies of donor management describe variation in practice and outcomes between clinicians, hospitals, and donation regions,^57^, ^58^, ^59^ routinely collected donation and transplantation data are insufficient to determine whether, and to what extent, innovations have been adopted and are effective in real-world settings. Although this challenge exists in many healthcare settings, the US donor management systems’ tripartite organization further complicates evidence translation because different stakeholders may have variable knowledge (and opinions of) new evidence and varying jurisdiction and interest in changing practice.

### Opportunities and next steps

Looking forward, there are several opportunities to accelerate generation and implementation of evidence in deceased donor management. First, to ensure adherence to ethical and regulatory best practice in donor management research, the National Academies of Sciences, Engineering, and Medicine recommend the establishment of centralized oversight structures.^5^ A Donor Research Oversight Committee working in close coordination with a specialty institutional review board for donor research may prioritize research questions, streamline multi-site approvals, and ensure appropriate ethical oversight for studies frequently classified as exempt from human subjects research.^60^ Second, stakeholder engagement must become a foundational aspect of donor research. To prioritize this need, dedicated funding for patient, family, and donor partners' participation in research governance, priority setting, and study conduct is critical to ensure that those most affected by donation decisions have a voice in shaping the research agenda.^61^, ^62^ Third, donor management research may further embrace recent innovations in clinical trial design.^35^ The simvastatin trial by Nykänen et al. exemplifies a methodologically rigorous approach by administering interventions after organ allocation to focus on graft quality rather than yield. By incorporating markers of systemic inflammation, detailed graft function assessments, and pathologic examinations alongside standard clinical outcomes, this trial design offers opportunities to collect Phase II data necessary to prioritize future resource-intensive clinical trials required to prove efficacy (as in the SIGNET protocol^54^). Fourth, in the United States, the growing use of donor care centers provides unprecedented infrastructure for adaptive platform trials, enabling the efficient testing of multiple interventions in standardized environments with consistent protocols and experienced staff,^14^, ^63^ donor care centers were key study sites in the donor hypothermia trial discussed above.^40^

Donor management has long relied on clinical protocols as implementation tools; these may be easily adapted to incorporate new findings. Recent work by Westphal et al^52^ expanded on the Malinoski’s foundational work over the last 15 years^48^, ^49^, ^50^ to improve care quality for potential DBD donors in Brazil. Ultimately the trial’s results were limited by adherence and adoption; however, the underlying principles are based on sound clinical evidence, and improved outcomes in cases of greatest intervention adherence demonstrated the efficacy of protocolized donor management. Implementation efforts that improve usability and promote adoption should continue rather than abandoning these structures.

### Limitations

We acknowledge several limitations to this narrative review. We did not conduct systematic review and limited our literature search to studies published in English. Many of the trials reported herein are limited by observational design and are thus inadequate to justify changes in practice at this time. The authorship team’s professional experience is limited to the US, the United Kingdom, and Italian donation systems and may therefore lack critical insights from other countries and regions. Additionally, we focused exclusively on DBD and did not address the growing practice of donation after circulatory death.^64^ Although evolving organ preservation and assessment techniques (normothermic regional perfusion and ex vivo machine perfusion) may fundamentally alter the importance of deceased donor management, we did not include evidence supporting these approaches as they fall beyond the scope of practice for most intensivists. Although we highlighted international variation in some published guidelines, a comprehensive comparison of donation and transplant outcomes between countries is beyond the scope of this work. Notwithstanding these limitations, we believe this review offers valuable insights into the current state of evidence and highlights critical knowledge gaps that, if addressed, offer opportunities for intensivists to improve donor management practices and outcomes for donors, patients, and transplant recipients.

### Conclusion

Despite significant interest and the pressing need to increase the supply and quality of organs to treat end-stage disease, in the last 10 years there have been fewer than ten multicenter randomized controlled trials conducted in potential thoracic organ DBD sufficient to meaningfully inform changes to practice. While work is ongoing, the pace of change is insufficient to address this critical public health challenge.

## Declaration of Generative AI and AI-Assisted Technologies in the Writing Process

During the preparation of this work, the authors used Claude Opus 4 [Anthropic. (2024). https://claude.ai] in order to improve language and readability. After using this tool/service, the authors reviewed and edited the content as needed and take full responsibility for the content of the publication.

## Conflicts of Interest Statement

The authors declare that they have no known competing financial interests or personal relationships that could have appeared to influence the work reported in this paper.
